# Supplementary material for: The Impact of Kefir Consumption on Inflammation, Oxidative Stress Status, and Metabolic-Syndrome-Related Parameters in Animal Models: A Systematic Review and Meta-Analysis
Source: Foods. 2025 Jun 12;14(12):2077. doi: 10.3390/foods14122077 (PMC12192021; doi:10.3390/foods14122077)
Supplement: Supplementary file 1 [file foods-14-02077-s001.zip › foods-3653423-supplementary.pdf]

Supplementary Table S1

Database search strategy for *in vivo* experiments investigating the beneficial effects of kefir consumption on metabolic syndrome.

Databases	Keywords	Results
PubMed	((kefir) AND (((rat) OR (mice)) OR (in vivo))) AND (((high sucrose diet) OR (high fat diet)) OR (high fructose diet))	34
SCOPUS	(TITLE-ABS-KEY (kefir)) AND ((TITLE-ABS-KEY ("in vivo") OR TITLE-ABS-KEY (rat) OR TITLE-ABS-KEY (mice))) AND ((TITLE-ABS-KEY ("high fat diet") OR TITLE-ABS-KEY ("high fructose diet") OR TITLE-ABS-KEY ("high sucrose diet")))	32
AMED	TI kefir AND AB metabolic syndrome AND AB (mice or rats or rodents)	1
LILACS	Title, abstract, subject: Kefir and rat or mice or rodent	53

Supplementary Table S2

Assessment of methodological quality in the included studies using the 10-item CAMARADES checklist

	Checklist items									
Author, Year	1	2	3	4	5	6	7	8	9	10
1. Akar et al., 2021	y	y	y	y	n	y	y	n	y	y
2. Akar et al., 2022	y	y	y	y	n	y	y	n	y	y
3. Angelis-Pereira et al., 2013	y	y	y	y	n	y	y	n	y	n
4. Bourrie et al., 2018	y	y	n	y	n	y	y	n	y	n
5. Bourrie et al., 2021	y	n	y	y	y	y	y	n	y	y
6. Bourrie et al., 2022	y	n	y	y	y	y	y	n	y	y
7. Chang et al., 2023	y	y	y	y	n	y	y	n	y	y
8. Chen et al., 2016	y	y	y	y	n	y	y	n	y	y
9. Chen et al., 2018	y	y	y	y	n	y	y	n	y	y
10. Chen et al., 2021	y	n	y	y	n	y	y	n	y	y
11. Cho et al., 2018	y	y	y	y	n	y	y	n	y	y
12. Choi et al., 2006	y	y	n	y	n	y	y	n	y	n
13. Choi et al., 2017	y	n	y	y	n	y	y	n	y	y
14. Ekici et al., 2022	y	n	y	y	n	y	y	n	y	y
15. Ekici et al., 2022	y	n	n	y	n	y	y	n	y	y
16. Gao et al., 2019	y	n	y	y	n	y	y	n	y	y
17. Gao et al., 2021	y	n	y	y	y	y	y	n	y	y
18. Hammami et al., 2022	y	y	y	y	n	y	y	n	y	y
19. Kim et al., 2017	y	n	y	y	n	y	y	n	y	y
20. Kim et al., 2017	y	n	y	y	n	y	y	n	y	y
21. Kim et al., 2021	y	n	y	y	n	y	y	n	y	y
22. Kwon et al., 2019	y	n	y	y	n	y	y	n	y	y

Supplementary Table S2

Assessment of methodological quality in the included studies using the 10-item CAMARADES checklist

	Checklist items									
Author, Year	1	2	3	4	5	6	7	8	9	10
23. Lim et al., 2017	y	y	n	y	n	y	y	n	y	y
24. Lin et al., 2016	y	n	n	y	n	y	y	n	y	n
25. Lin et al., 2020	y	n	n	y	n	y	y	n	y	n
26. Nurliyani et al., 2022	y	n	n	y	n	y	y	n	y	y
27. Salah et al., 2023	y	y	y	y	n	y	y	n	y	y
28. Santanna et al., 2017	y	y	n	y	n	y	y	n	y	n
29. Seo et al., 2022	y	n	y	y	n	y	y	n	y	y
30. Seo et al., 2020	y	y	n	y	n	y	y	n	y	y
31. Susanti et al., 2022	y	n	n	y	n	y	y	n	y	y
32. Talib et al., 2023	y	y	y	y	n	y	y	n	y	y
33. Tarakci et al., 2022	y	n	y	y	n	y	y	n	y	y
34. Tung et al., 2018	y	y	n	y	n	y	y	n	y	y
35. Tung et al., 2020	y	y	y	y	n	y	y	n	y	y
36. Youn et al., 2022	y	n	y	y	n	y	y	n	y	y
37. Zheng et al., 2024	y	y	n	y	n	y	y	n	y	y
38. Zubiría et al., 2017	y	y	y	y	n	y	y	n	y	y

Studies fulfilling the criteria of: 1= the peer-reviewed publication; 2=control of temperature; 3=random allocation to treatment or control; 4=blinded induction of metabolic syndrome; 5=blinded assessment of outcome; 6=use of co-interventions/co-morbid without significant intrinsic protective effect toward metabolic syndromes; 7=appropriate animal model (age, sex, species, strain); 8=sample size calculation; 9=compliance with animal welfare regulations; and 10=statement of potential conflict of interests.

Supplementary Table S3

Risk of bias assessment of the included studies using the SYRCLE tool.

Author Year	Types of bias and check items									
	Selection bias			Performance bias		Detection bias		Attrition bias	Reporting bias	Other sources of bias
	SB1	SB2	SB3	PB1	PB2	DB1	DB2			
1. Akar et al., 2021	Low	Unclear	High	Low	High	High	High	Low	Unclear	Low
2. Akar et al., 2022	Low	Low	High	Unclear	High	High	High	Unclear	Unclear	Low
3. Angelis-Pereira et al., 2013	Low	Unclear	High	Unclear	High	High	High	Unclear	Low	Low
4. Bourrie et al., 2018	High	Low	High	Unclear	High	High	High	Unclear	Unclear	Low
5. Bourrie et al., 2021	Low	Unclear	Low	Low	Low	High	Low	Low	Unclear	Low
6. Bourrie et al., 2022	Low	Unclear	Low	Low	Low	High	Unclear	Low	Unclear	Low
7. Chang et al., 2023	Low	Unclear	High	Unclear	High	High	High	High	Low	Low
8. Chen et al., 2016	Low	Low	High	Unclear	High	High	High	Low	Low	Low
9. Chen et al., 2018	Low	Low	High	Unclear	High	High	High	Unclear	Unclear	Low
10. Chen et al., 2021	Low	Low	High	Unclear	High	High	High	Unclear	Unclear	Low
11. Cho et al., 2018	Low	Unclear	High	Unclear	High	High	High	High	Unclear	Low
12. Choi et al., 2006	High	Unclear	High	Unclear	High	High	High	Unclear	Unclear	Low
13. Choi et al., 2017	Low	Low	High	Unclear	High	High	High	Unclear	Low	Low

Supplementary Table S3

Risk of bias assessment of the included studies using the SYRCLE tool.

Author Year	Types of bias and check items									
	Selection bias			Performance bias		Detection bias		Attrition bias	Reporting bias	Other sources of bias
	SB1	SB2	SB3	PB1	PB2	DB1	DB2			
14. Ekici et al., 2022	Low	Unclear	High	Unclear	High	High	High	Unclear	Unclear	Low
15. Ekici et al., 2022	High	Unclear	High	Unclear	High	High	High	Unclear	Unclear	Low
16. Gao et al., 2019	Low	Low	High	Unclear	High	High	High	Unclear	Low	Low
17. Gao et al., 2021	Low	Low	High	Unclear	High	Low	High	Unclear	Low	Low
18. Hammami et al., 2022	Low	Low	High	Low	High	High	High	Unclear	Low	Low
19. Kim et al., 2017	Low	Low	High	Unclear	High	High	High	Unclear	Unclear	Low
20. Kim et al., 2017	Low	Unclear	High	Unclear	High	High	Low	Low	Unclear	Low
21. Kim et al., 2021	Low	Unclear	High	Unclear	High	High	High	Low	Unclear	Low
22. Kwon et al., 2019	Low	Unclear	High	Unclear	High	High	High	Low	Unclear	Low
23. Lim et al., 2017	High	Low	High	Unclear	High	High	High	Unclear	Unclear	Low
24. Lin et al., 2016	High	Low	High	Unclear	High	High	High	Low	Low	Low
25. Lin et al., 2020	High	Unclear	High	Unclear	High	High	High	Unclear	Unclear	Low
26. Nurliyani et al., 2022	High	Unclear	High	Unclear	High	High	High	Unclear	Unclear	Low
27. Salah et al., 2023	Low	Low	High	Unclear	High	High	High	Low	Low	Low

Supplementary Table S3

Risk of bias assessment of the included studies using the SYRCLE tool.

Author Year	Types of bias and check items									
	Selection bias			Performance bias		Detection bias		Attrition bias	Reporting bias	Other sources of bias
	SB1	SB2	SB3	PB1	PB2	DB1	DB2			
28. Santanna et al., 2017	High	Unclear	High	Unclear	High	High	Low	Unclear	Unclear	Low
29. Seo et al., 2022	Low	Unclear	High	Unclear	High	High	High	Unclear	Unclear	Low
30. Seo et al., 2020	High	Unclear	High	Unclear	High	High	High	Unclear	Unclear	Low
31. Susanti et al., 2022	High	Unclear	High	Unclear	High	High	High	Unclear	Unclear	Low
32. Talib et al., 2023	Low	Low	High	Unclear	High	High	High	Unclear	Unclear	Low
33. Tarakci et al., 2022	Low	Low	High	Low	High	High	High	Low	Low	Low
34. Tung et al., 2018	High	Low	High	Unclear	High	High	High	Low	Low	Low
35. Tung et al., 2020	Low	Unclear	High	Unclear	High	High	High	Low	Low	Low
36. Youn et al., 2022	Low	Unclear	High	Unclear	High	High	High	High	Unclear	Low
37. Zheng et al., 2024	High	Unclear	High	Unclear	High	High	High	Unclear	Low	Low
38. Zubiría et al., 2017	Low	Low	High	Unclear	High	High	High	Low	Low	Low

Supplementary Table S4

General characteristics of included studies

Author	Year	Country	Characteristics of rodents used				
			No. of animal		Types of rodents	Age (wk.)	Sex
			Total	per group			
1. Akar et al.	2021	Turkey	27	9	Wistar	3	Male
2. Akar et al.	2022	Turkey	NM	6-8	Wistar	3	Male
3. Angelis-Pereira et al.	2013	Brazil	30	6	Wistar	NM	Male
4. Bourrie et al.	2018	Canada	56	8	C57BL/6	8	Female
5. Bourrie et al.	2022	Canada	80	4	C57BL/6	8	Male/ Female
6. Bourrie et al.	2021	Canada	40	4	C57BL/6	8	Female
7. Chang et al.	2023	Taiwan	30	6	C57BL/6 (B6) $ApoE^{-/-}$	7	Male
8. Chen et al.	2016	Taiwan	48	8	C57BL/6Jnarl	6	Male
9. Chen et al.	2021	Taiwan	50	10	C57BL/6	7	Male
10. Chen et al.	2018	Taiwan	60	12	Sprague Dawley	6	Male
11. Cho et al.	2018	South Korea	60	10	C57BL/6J	22	Male
12. Choi et al.	2017	South Korea	48	8	C57BL/6J	4	Male
13. Choi et al.	2006	South Korea	24	8	Sprague Dawley	NM	Male
14. Ekici et al.	2022	Turkey	NM	6-8	Wistar	4	Male
15. Ekici et al.	2022	Turkey	NM	6-12	Wistar	4	Male
16. Gao et al.	2019	China	15	5	Sprague Dawley	8	Female
17. Gao et al.	2021	China	20	5	Sprague Dawley	8	Female
18. Hammami et al.	2022	Tunisia	24	6	Rat	10	Female
19. Kim et al.	2017	South Korea	40	4-8	C57BL/6	4	Male
20. Kim et al.	2017	South Korea	20	2	C57BL/6	4	Male

Supplementary Table S4

General characteristics of included studies

Author	Year	Country	Characteristics of rodents used				
			No. of animal		Types	Age	Sex
			Total	per group	of rodents	(wk.)	
21. Kim et al.	2021	South Korea	30	10	C57BL/6J	4	Male
22. Kwon et al.	2019	South Korea	40	10	C57BL/6J	4	Male
23. Lim et al.	2017	South Korea	36	9	C57BL/6J	4	Male
24. Lin et al.	2016	Taiwan	40	10	C57BL/6J	7	Male
25. Lin et al.	2020	Taiwan	40	10	C57BL/6J	7	Male
26. Nurliyani et al.	2022	Indonesia	30	6	Sprague Dawley	8-12	Male
27. Salah et al.	2023	Egypt	24	8	Wistar	6	Male
28. Santanna et al.	2017	Brazil	33	6-9	$LDLr^{-/-}$	6	Male
29. Seo et al.	2022	South Korea	50	10	C57BL/6	4	Male
30. Seo et al.	2020	Canada	30	10	C57BL/6J	4	Male
31. Susanti et al.	2022	Indonesia	21	7	BALB/c	8-12	NM
32. Talib et al.	2023	Malaysia	32	8	C57BL/6	4-5	Male
33. Tarakci et al.	2022	Turkey	35	3/ 4	Wistar	8	Male/ Female
34. Tung et al.	2020	Taiwan	40	8	$ApoE^{-/-}$ C57BL/6	7	Male
35. Tung et al.	2018	Taiwan	32	8	Sprague Dawley	6	Male
36. Youn et al.	2022	South Korea	50	10	C57BL/6J	3	Male
37. Zheng et al.	2024	South Korea	90	10	C57BL/6J	4	Male
38. Zubiría et al.	2017	Argentina	30	15	Swiss	4	Male

*NM: Not Mentioned

Supplementary Table S5

Strategies for inducing metabolic syndrome in animal models for included studies.

Author, Year	Methods used (duration)	The construction of the induction		
		Diet	Chemical	Genetic
1. Akar et al., 2021	20% fructose solution (w/v) in drinking water, ad libitum (6 days)	√	–	–
2. Akar et al., 2022	20% fructose solution (w/v) in drinking water, daily (15 weeks)	√	–	–
3. Angelis-Pereira et al., 2013	Hypercholesterolemic diet (0.5% cholesterol and 3.5% hydrogenate vegetable fat) (3 weeks)	√	–	–
4. Bourrie et al., 2018	HFD (40% energy from fat and 1.25% cholesterol), ad libitum (12 weeks)	√	–	–
5. Bourrie et al., 2022	HFD (40 % energy from fat and 1.25% cholesterol by weight), ad libitum (8 weeks)	√	–	–
6. Bourrie et al., 2021	HFD (40 % energy from fat and 1.25% cholesterol) (8 weeks)	√	–	–
7. Chang et al., 2023	Atherogenic diet (4.05 kcal/g) with 1.25% cholesterol, ad libitum (6 weeks)	√	–	√
8. Chen et al., 2016	30% fructose corn syrup in drinking water, ad libitum (2 weeks)	√	–	–
9. Chen et al., 2021	(1) HFD (60% energy from fat), daily (10 weeks) (2) WD (17%, 43%, and 40% energy from protein, carbohydrates, and fat, respectively), daily (10 weeks)	√	–	–

Supplementary Table S5

Strategies for inducing metabolic syndrome in animal models for included studies.

Author, Year	Methods used (duration)	The construction of the induction		
		Diet	Chemical	Genetic
10. Chen et al., 2018	HFD (60% energy from fat diet), daily (12 weeks)	√	–	–
11. Cho et al., 2018	HFD (46% energy from fat), ad libitum (5 weeks)	√	–	–
12. Choi et al., 2017	HFD (45% energy from fat), ad libitum (4 weeks)	√	–	–
13. Choi et al., 2006	HFD (10% lactobacillus ferment), ad libitum (4 weeks)	√	–	–
14. Ekici et al., 2022	High fructose corn syrup 20% solution (w/v) in drinking water, ad libitum (8 weeks)	√	–	–
15. Ekici et al., 2022	High fructose corn syrup 20% solution (w/v) in drinking water, ad libitum (8 weeks)	√	–	–
16. Gao et al., 2019	HFD with (60% energy from fat), (8 weeks)	√	–	–
17. Gao et al., 2021	HFD (60% energy from fat), daily (8 weeks)	√	–	–
18. Hammami et al., 2022	HFD with 0.001 mL/g body weight of semi-skimmed cow milk (8 weeks)	√	–	–
19. Kim et al., 2017	HFD (60% energy from fat), twice daily (9:00 and 18:00 h) (12 weeks)	√	–	–
20. Kim et al., 2017	HFD (60% energy from fat), daily (6 weeks)	√	–	–
21. Kim et al., 2021	HFD, oral administration, (6 weeks)	√	–	–
22. Kwon et al., 2019	HFD with 10% GSF, ad libitum (9 weeks)	√	–	–

Supplementary Table S5

Strategies for inducing metabolic syndrome in animal models for included studies.

Author, Year	Methods used (duration)	The construction of the induction		
		Diet	Chemical	Genetic
23. Lim et al., 2017	HFD with 5% MCC, daily (4 weeks)	√	–	–
24. Lin et al., 2016	HFD (60% energy from fat) with PBS, daily (8 weeks)	√	–	–
25. Lin et al., 2020	HFD (60% kcal from fat) daily (8 weeks)	√	–	–
26. Nurliyani et al., 2022	HFHF diet (321.6 g of Fructose) (6 weeks)	√	–	–
27. Salah et al., 2023	HFHS (70% standard chow, 20% lard, 10% sucrose, 1% cholesterol, and 0.25% cholic acid), ad libitum (9 or 12 weeks)	√	–	–
28. Santanna et al., 2017	WD (17.9% protein, 7% fat, 4.8% fiber, 7% moisture and 4.2% ash), oral administration (4 weeks)	√	–	√
29. Seo et al., 2022	HFD (46% energy from fat), ad libitum, (6 weeks)	√	–	–
30. Seo et al., 2020	HFHF diet (~47% and ~36.4-36.7 energy from fat and carbohydrate, respectively), ad libitum (8 weeks)	√	–	–
31. Susanti et al., 2022	HFHF (Standard food mixed with liquid margarine at 0.238 g/mouse/day and 2 mL/mouse/day of fructose solution), (3 weeks)	√	–	–
32. Talib et al., 2023	HFD (45% energy from fat), (8 weeks)	√	√	–
33. Tarakci et al., 2022	HFD (60% energy from fat), daily, (8 weeks)	√	–	–

Supplementary Table S5

Strategies for inducing metabolic syndrome (MetS) in animal models for included studies.

Author, Year	Methods used (duration)	The construction of the induction		
		Diet	Chemical	Genetic
34. Tung et al., 2020	Atherogenic HFD (61.6% fat, 20.3% carbohydrates, and 18.1% protein), daily (12 weeks)	√	–	√
35. Tung et al., 2018	HFD (8% (w/w) soybean oil+ 44% (w/w) sweetened milk), daily (6 weeks)	√	–	–
36. Youm et al., 2022	HFD (45% energy from fat), ad libitum (1 week)	√	–	–
37. Zheng et al., 2024	HFD (60% energy from fat), ad libitum (8 weeks)	√	–	–
38. Zubiría et al., 2017	FRD (20% fructose (w/v) in drinking water), (6 weeks)	√	–	–

*HFD; High-fat diet, HFHF; High-fat high fructose, SCD; Standard chow diet, WD; Western diet, HFHS; High-fat, high sucrose, FRD; fructose-rich diet.

Supplementary Table S6

The summary of outcomes related to parameters associated with metabolic syndrome, inflammation, and oxidative stress highlights the effects of kefir and its active components in rodent models.

Author, Year	Metabolic syndrome related parameters	Inflammatory markers	Oxidative stress markers
1. Akar et al., 2021	↓WG, ↓TG, ↑HDL, ↓PG, ↓PI	↓TNF-α, ↓IL-1β	-
2. Akar et al., 2022	↓WG, ↓TG, ↑HDL, ↓PG, ↓PI	-	-
3. Angelis-Pereira et al., 2013	↓LDL-C, ↓TG, ↑HDL-C	-	-
4. Bourrie et al., 2018	↓TG, ↓TC, ↓TG, ↓HDL-C	-	-
5. Bourrie et al., 2022	↓WG, ↓TC, ↓, ↓HDL-C	-	-
6. Bourrie et al., 2021	↓WG, ↓TC, ↓HDL-C	-	-
7. Chang et al., 2023	↓WG, ↓TC, ↑TG	↓TNF-α	↓MDA, ↓ox-LDL
8. Chen et al., 2016	↓WG, ↓TC, ↓TG	-	-
9. Chen et al., 2021	↓WG, ↓TG, ↓PG	-	-
10. Chen et al., 2018	↓WG, ↓PG, ↓PI	-	-
11. Cho et al., 2018	↓WG, ↓TC, ↓TG, ↓LDL-C, ↓HDL-C, ↓VDL-C, ↓PG, ↓PI	-	-
12. Choi et al., 2017	↓WG, ↓TC, ↓TG, ↓LDL-C, ↑HDL-C	-	-
13. Choi et al., 2006	↓WG, ↓TC, ↓TG, ↑HDL-C	-	-
14. Ekici et al., 2022	↓WG, ↓TC, ↓TG, ↓VDL-C ↓PG, ↓PI	-	-
15. Ekici et al., 2022	↓WG, ↓TC, ↓TG, ↓VDL-C ↓PG, ↓PI	-	-
16. Gao et al., 2019	↓WG, ↓TC, ↓TG, ↓LDL-C, ↓HDL-C	-	-

Supplementary Table S6

The summary of outcomes related to parameters associated with metabolic syndrome, inflammation, and oxidative stress highlights the effects of kefir and its active components in rodent models.

Author, Year	Metabolic syndrome related parameters	Inflammatory markers	Oxidative stress markers
17. Gao et al., 2021	↓WG, ↓TC, ↓TG, ↓LDL-C, ↓HDL-C	-	-
18. Hammami et al., 2022	↓WG, ↓TC, ↓TG, ↑LDL-C, ↑HDL-C, ↓PG	-	-
19. Kim et al., 2017	↓WG, ↓TC, ↓TG, ↓LDL-C, ↑HDL-C	-	-
20. Kim et al., 2017	↓WG, ↓TC, ↓TG, ↓LDL-C, ↓HDL-C	↑TNF-α, ↓IL-6, ↑ IL-1β	-
21. Kim et al., 2021	↓WG, ↓TC, ↓TG, ↓LDL-C, ↓HDL-C, ↓PG, ↓PI	-	-
22. Kwon et al., 2019	↓WG, ↓ TC, ↓TG, ↓LDL-C, ↓VLDL-C, ↓PG, ↓PI	-	-
23. Lim et al., 2017	↓WG, ↓TC, ↓LDL-C, ↓VDL-C ↑HDL-C, ↓VLDL-C, ↓PG, ↓PI	-	-
24. Lin et al., 2016	↓BW, ↓TC, ↓TG, ↓LDL-C, HDL-C, ↓PG, ↓PI	↓IL-6	-
25. Lin et al., 2020	↓WG	-	-
26. Nurliyani et al., 2022	-	↓TNF	-
27. Salah et al., 2023	↓TC, ↓TG, ↓LDL-C, ↑HDL-C	-	-
28. Santanna et al., 2017	↓TC, ↑LDL-C	↓TNF-α, ↓IL-6	-
29. Seo et al., 2022	↓WG, ↓TC, ↓TG, ↓LDL-C	-	-

Supplementary Table S6

The summary of outcomes related to parameters associated with metabolic syndrome, inflammation, and oxidative stress highlights the effects of kefir and its active components in rodent models.

Author, Year	Metabolic syndrome related parameters	Inflammatory markers	Oxidative stress markers
30. Seo et al., 2020	↓WG, ↓TC, ↓TG, ↓LDL-C, ↓HDL-C, ↑PG	-	-
31. Susanti et al., 2022	↓PG	-	-
32. Talib et al., 2023	↑WG, ↓TC, ↓TG, ↓LDL-C, ↑HDL-C	-	-
33. Tarakci et al., 2022	↓WG, ↓PG	-	-
34. Tung et al., 2020	↓TC, ↓TG, ↑LDL-C	↓TNF-α, ↓IL-1β	-
35. Tung et al., 2018	↓WG, ↑TC, ↑TG, ↓LDL-C, ↑HDL-C	-	-
36. Youm et al., 2022	↓WG, ↓TC, ↓TG, ↑LDL-C, ↑HDL-C	-	-
37. Zheng et al., 2024	↓WG, ↓TC, ↓TG, ↓PG	-	-
38. Zubiría et al., 2017	↓WG, ↓TG, ↓PG	-	-

* WG: Weight gain, TC: Total cholesterol, TG: Triglyceride, LDL-C: Low-density lipoprotein-cholesterol, HDL-C: High-density lipoprotein-cholesterol, VLDL-C: Very low-density lipoprotein-cholesterol, PG: Plasma glucose, PI: plasma insulin, TNF-α: Tumor necrosis factor-α, IL-6: Interleukin-6, IL-1β: Interleukin-1β

Supplementary Table S7

Evaluating outcomes associated with metabolic syndrome parameters highlights the effects of kefir and its active components in rodent models.

Author, Year	Metabolic-related parameters (mean±SD)						
(Tested group)	WG (g)	TC (mg/dL)	TG (mg/dL)	LDL-C (mg/dL)	HDL-C (mg/dL)	PG (mg/dL)	PI (ng/mL)
1. Akar et al., 2021							
Control	365.4±8.9	-	114.4±5.3	-	23.1±3.7	106.6±0.7	1.6±0.4
Fructose	374.0 ± 13.9	-	323.8±10.2	-	25.5±4.3	154.3±8.0	3.4±0.7
Fructose+kefir	354.9 ± 7.9	-	133.7±15.3	-	26.8±1.0	141.8±8.4	1.8±0.2
2. Akar et al., 2022							
Control	365.4±8.9	-	114.4±5.3	-	23.1±3.7	106.6±0.7	1.6±0.4
Fructose	374.0±13.9	-	323.8±10.2	-	25.5±4.3	154.3±8.0	3.4±0.7
Fructose + kefir	354.9±7.9	-	133.7±15.3	-	26.8±1.0	141.8±8.4	1.8±0.2
3. Angelis-Pereira al., 2013							
Control	-	-	90.0±15.2	-	44.0±5.1	-	-
HCLD	-	-	108.9±8.5	-	54.37±4.7	-	-
HCLD+kefir	-	-	67.1±6.4	-	64.9±4.2	-	-

Supplementary Table S7

Evaluating outcomes associated with metabolic syndrome parameters highlights the effects of kefir and its active components in rodent models.

Author, Year	Metabolic-related parameters (mean±SD)						
(Tested group)	WG (g)	TC (mg/dL)	TG (mg/dL)	LDL-C (mg/dL)	HDL-C (mg/dL)	PG (mg/dL)	PI (ng/mL)
4. Bourrie et al., 2018							
Control	-	195±10.2	320.0±3.0	-	36.0±5.5	-	-
HFD+C-Kefir	-	190±6.5	260.0±2.5	-	35.0±5.5	-	-
HFD+ICK	-	110±1.9	220.0±3.5	-	23.0±2.0	-	-
HFD+IR9	-	140±10.2	290.0±2.0	-	39.0±4.9	-	-
HFD+IR10	-	108±2.1	255.0±3.0	-	29.5±1.2	-	-
HFD+GER2	-	145±6.3	300.0±1.5	-	30.0±4.5	-	-
5. Bourrie et al., 2022							
Control	12.0±2.0 (M)	350.0±1.0	-	-	65.0±0.8	-	-
	7.5±2.0 (F)	160.0±1.5	-	-	31.0±1.5	-	-
HFD+PK1	8.0±1.8 (M)	225.0±1.0	-	-	64.0±1.2	-	-
	6.2±1.5 (F)	80.0±2.0	-	-	27.0±0.5	-	-
HFD+CFK	7.0±1.5 (M)	200.0±2.5	-	-	55.0±0.7	-	-
	8.3±1.8 (F)	90.0±1.8	-	-	28.0±2.0	-	-
HFD+HK	6.5±2.0 (M)	220.0±2.5	-	-	56.5±0.3	-	-
	6.5±4.5 (F)	120±1.0	-	-	26.5±0.9	-	-

Supplementary Table S7

Evaluating outcomes associated with metabolic syndrome parameters highlights the effects of kefir and its active components in rodent models.

Author, Year	Metabolic-related parameters (mean±SD)						
(Tested group)	WG (g)	TC (mg/dL)	TG (mg/dL)	LDL-C (mg/dL)	HDL-C (mg/dL)	PG (mg/dL)	PI (ng/mL)
6. Bourrie et al., 2021							
HFD+C-Kefir	12.0±3.0	4504.5±5.0	-	-	756.7±1.5	-	-
HFD+ICK	9.5±5.5	3243.2±1.0	-	-	630.6±2.5	-	-
HFD+P-Kefir	8.0±2.0	3063.1±1.5	-	-	756.7±8.5	-	-
HFD+PNY	11.5±6.0	3961.0±3.5	-	-	621.6±2.0	-	-
HFD+PNL	12.5±4.5	4054±3.0	-	-	720.7±1.7	-	-
7. Chang et al., 2023							
Control	5.1±1.8	1896±181.7	26.7±8.4	-	-	-	-
AD+KPs-L	4.5±1.2	1418±292.3	27.7±12.3	-	-	-	-
AD+KPs-H	5.1±1.8	1409±301.9	40.8±15.9	-	-	-	-
8. Chen et al., 2016							
Control	4.2±0.2	73.8±0.8	27.9±1.2	-	-	-	-
Fructose+ KPs-L	4.2±0.23	71.0±1.5	22.4±1.3	-	-	-	-
Fructose+ KPs-M	4.1±0.3	69.1±2.3	22.5±1.2	-	-	-	-
Fructose+ KPs-H	3.1±0.1	71.9±2.0	22.3±0.7	-	-	-	-
Fructose +CFM	4.3±0.4	75.5±2.8	24.8±1.4	-	-	-	-

Supplementary Table S7

Evaluating outcomes associated with metabolic syndrome parameters highlights the effects of kefir and its active components in rodent models.

Author, Year	Metabolic-related parameters (mean±SD)						
(Tested group)	WG (g)	TC (mg/dL)	TG (mg/dL)	LDL-C (mg/dL)	HDL-C (mg/dL)	PG (mg/dL)	PI (ng/mL)
9. Chen et al., 2021							
Control (HFD)	18.0±4.0	-	80.0±3.0	-	-	405.0±3.0	-
HFD+ABK	13.0±2.5	-	65.0±2.0	-	-	270.0±2.0	-
Control (WD)	13.5±4.0	-	110.0±8.5	-	-	380.0±1.7	-
WD+ABK	11.0±4.0	-	109.0±3.5	-	-	385.0±5.0	-
10. Chen et al., 2018							
Control	425.3±10.5	-	-	-	-	215.4±4.4	3.0±0.3
HFD+ APS1-L	427.9±10.6	-	-	-	-	206.7±16.4	2.5±0.2
HFD+ APS1-M	366.9±14.1	-	-	-	-	152.2±9.2	2.3±0.2
HFD+ APS1-H	387.3±11.8	-	-	-	-	145.6±12.7	2.0±0.3
11. Cho et al., 2018							
Control	13.3±0.5	266±13.4	51.2±5.1	28.8±3.6	94.4±8.1	172±6.2	2.7±0.3
GSFL+LAB	5.2±1.0	196±6.4	29.2±2.5	21.0±1.9	93.2±5.8	143±4.9	1.6±0.2
GSFH+LAB	-1.0±0.4	161±10.1	31.2±2.2	19.8±1.3	90.7±4.5	136±5.7	0.8±0.1
LAB	7.9±0.6	233±15.7	37.1±3.6	22.6±1.8	77.1±8.4	157±10	1.1±0.3

Supplementary Table S7

Evaluating outcomes associated with metabolic syndrome parameters highlights the effects of kefir and its active components in rodent models.

Author, Year	Metabolic-related parameters (mean±SD)						
(Tested group)	WG (g)	TC (mg/dL)	TG (mg/dL)	LDL-C (mg/dL)	HDL-C (mg/dL)	PG (mg/dL)	PI (ng/mL)
12. Choi et al., 2017							
Control	36.0±0.5	183±15.6	68.5±3.9	20.5±2.8	111.5±4.6	-	-
HFD-LK	32.5±1.0	133.3±6.0	66.2±3.3	12.0±1.6	128.4±2.8	-	-
HFD-HK	26.5±0.5	133.2±3.1	49.9±2.0	12.3±1.1	125.1±3.1	-	-
13. Choi et al., 2006							
Control	210.0±47.6	123.7±4.6	58.2±4.8	-	56.7±4.8	-	-
HFD+Lactic-F	148.4±28.0	87.5±9.3	42.5±2.0	-	68.5±5.8	-	-
14. Ekici et al., 2022							
Control	365±9.0	67.1±3.1	179±1.0	-	-	106±4.0	1.9±0.05
Kefir	322±3.0	59.6±3.8	146±2.1	-	-	79±1.6	0.7±0.07
HFCS+kefir	335±2.0	55.3±2.4	160±1.5	-	-	87±0.7	0.9±0.06
15. Ekici et al., 2022							
Control	365±9.0	67.1±3.1	179±1.0	-	-	106±4.0	1.9±0.05
Kefir	322±3.0	59.6±3.8	146±2.1	-	-	79±1.6	0.7±0.07
HFCS+kefir	335±2.0	55.3±2.4	160±1.5	-	-	87±0.7	0.9±0.06

Supplementary Table S7

Evaluating outcomes associated with metabolic syndrome parameters highlights the effects of kefir and its active components in rodent models.

Author, Year	Metabolic-related parameters (mean±SD)						
(Tested group)	WG (g)	TC (mg/dL)	TG (mg/dL)	LDL-C (mg/dL)	HDL-C (mg/dL)	PG (mg/dL)	PI (ng/mL)
16. Gao et al., 2019							
Control	110±1.5	85.8±15.5	66.4±14.2	3.8±1.2	62.6±10.8	-	-
HFD+TKM	90±1.0	73.5±7.4	41.6±5.3	3.5±0.4	55.3±5.0	-	-
17. Gao et al., 2021							
Control	93±0.5	92.8±2.5	72.6±0.5	3.9±1.3	67.3±5.5	-	-
HFD+TKM1	87±0.3	81.2±0.5	44.3±0.2	3.9±1.3	54.1±0.01	-	-
HFD+TKM2	45±4.5	73.5±0.7	62.9±0.7	3.5±0.5	49.9±2.5	-	-
18. Hammami et al., 2022							
Control	220±6.4	100±0.1	128.4±0.1	13.6±0.2	19.1±0.05	98.0±1.5	-
HFD+ kefir milk	138±6.4	90.0±0.2	102.8±0.2	15.3±0.2	23.2±0.1	95.0±2.5	-
19. Kim et al., 2017							
Control	16.0±0.3	230.0±0.1	38.0±0.1	19.0±0.05	99.0±0.2	-	-
HFD+DH4	13.5±0.2	245.0±0.2	42.0±0.2	17.0±0.3	110.0±0.1	-	-
HFD+DH5	11.3±0.3	195.0±0.1	35.0±0.1	16.0±0.2	90.0±0.2	-	-
HFD+DH7	13.7±0.2	210.0±0.1	36.0±0.1	17.0±0.2	100.0±0.1	-	-

Supplementary Table S7

Evaluating outcomes associated with metabolic syndrome parameters highlights the effects of kefir and its active components in rodent models.

Author, Year	Metabolic-related parameters (mean±SD)						
(Tested group)	WG (g)	TC (mg/dL)	TG (mg/dL)	LDL-C (mg/dL)	HDL-C (mg/dL)	PG (mg/dL)	PI (ng/mL)
20. Kim et al., 2017							
Control	22.1±0.9	138.0±3.0	22.0±1.0	20.0±0.5	70.0±2.0	-	-
Kefir	16.2±1.8	115.0±5.0	18.0±1.5	5.0±1.0	60.0±4.0	-	-
21. Kim et al., 2021							
Control	11.5±0.5	121.0±4.8	42.3±3.5	13.9±1.3	70.3±2.3	112.0±4.0	0.5±0.04
HFD+SDH5	10.0±1.0	96.0±4.3	32.5±2.8	7.5±1.4	70.2±2.8	107.0±3.0	0.3±0.1
HFD+SLCM8	10.2±0.5	106.0±5.1	30.3±1.1	15.3±2.5	68.4±2.1	110.0±2.8	0.3±0.01
22. Kwon et al., 2019							
Control	-	-	-	-	-	172.0±6.2	2.7±0.1
HFD+GSF+LAB	-	-	-	-	-	136.0±5.7	0.8±0.02
HFD+LAB	-	-	-	-	-	157±10.1	1.05±0.1
23. Lim et al., 2017							
Control	10.5±1.1	152.0±9.3	-	40.5±4.7	110.0±3.9	132.6±12.1	1.3±0.1
HFD+EPS	8.5±0.5	140.5±7.4	-	32.5±4.0	100.0±3.4	121.4±10.9	1.1±0.1
HFD+Res	9.4±0.8	142.0±8.6	-	30.0±4.3	112.5±3.6	126.5±11.6	1.2±0.1

Supplementary Table S7

Evaluating outcomes associated with metabolic syndrome parameters highlights the effects of kefir and its active components in rodent models.

Author, Year	Metabolic-related parameters (mean±SD)						
(Tested group)	WG (g)	TC (mg/dL)	TG (mg/dL)	LDL-C (mg/dL)	HDL-C (mg/dL)	PG (mg/dL)	PI (ng/mL)
24. Lin et al., 2016							
Control	9.9±1.6	158.8±10.7	78.6±31.8	19.5±3.6	146.1±10.2	190±10.0	0.0028±0.0009
HFD+M1	12.1±2.9	171.9±22.8	75.8±13.1	20.9±5.9	157.1±23.2	220±15.0	0.0032±0.001
HFD+APS1	6.8±2.6	148.5±21.3	60.6±17.8	13.5±4.7	136.3±17.4	175±10.0	0.0022±0.0007
25. Lin et al., 2020							
Control	30.0±2.0	-	-	-	-	-	-
HFD+M1	35.0±2.5	-	-	-	-	-	-
HFD+APS1	25.0±1.5	-	-	-	-	-	-
26. Nurliyani et al., 2022							
Control	-	-	-	-	-	118.9±11.3	-
HFHF+P-kefir	-	-	-	-	-	114.3±18.8	-
HFHF+S-kefir	-	-	-	-	-	105.1±11.9	-
HFHF+SIM	-	-	-	-	-	80.4±11.9	-

Supplementary Table S7

Evaluating outcomes associated with metabolic syndrome parameters highlights the effects of kefir and its active components in rodent models.

Author, Year	Metabolic-related parameters (mean±SD)						
(Tested group)	WG (g)	TC (mg/dL)	TG (mg/dL)	LDL-C (mg/dL)	HDL-C (mg/dL)	PG (mg/dL)	PI (ng/mL)
27. Salah et al., 2023							
Control (NASH)	-	181.9±3.5	141.5±10.1	123.5±1.5	30.1±2.4	-	-
Early kefir	-	99.0±9.3	73.5±3.7	34.1±11.3	50.2±3.0	-	-
Late kefir	-	126.7±1.6	94.5±4.9	47.3±4.2	60.5±2.3	-	-
28. Santanna et al., 2017							
Control	-	622.0±92.0	-	46.0±9.0	-	-	-
HFD+milk	-	572.0±97.0	-	38.0±8.0	-	-	-
HFD+kefir	-	554.0±10.2	-	50.0±7.0	-	-	-
29. Seo et al., 2022							
Control	12.5±0.6	121.0±4.8	44.1±3.5	13.9±1.3	-	-	-
HFD+EPS	12.0±0.5	110.0±4.3	37.0±1.9	9.2±1.2	-	-	-
HFD+SLP	10.0±0.4	106.0±5.1	30.3±1.1	15.3±2.5	-	-	-
30. Seo et al., 2020							
Control	13.5±1.2	136.0±8.8	99.0±8.9	17.0±0.7	79.0±2.9	203.0±18.6	-
HFFrD+HLAB	6.5±0.6	118.0±4.7	72.0±4.7	18.0±2.4	68.0±6.4	209.0±21.6	-

Supplementary Table S7

Evaluating outcomes associated with metabolic syndrome parameters highlights the effects of kefir and its active components in rodent models.

Author, Year (Tested group)	Metabolic-related parameters (mean±SD)						
	WG (g)	TC (mg/dL)	TG (mg/dL)	LDL-C (mg/dL)	HDL-C (mg/dL)	PG (mg/dL)	PI (ng/mL)
31. Susanti et al., 2022							
Control	-	-	-	-	-	122.7±12.3	-
HFHFD+kefir	-	-	-	-	-	103.2±8.4	-
32. Talib et al., 2023							
Control	8.0±1.6	33.6±0.7	42.6±1.8	3.5±0.4	14.3±0.7	-	-
HFD+L-kefir	11.0±1.1	22.8±1.2	15.2±0.9	2.7±0.4	15.8±0.7	-	-
HFD+H-kefir	12.0±1.3	22.4±4.6	14.8±3.5	2.3±0.4	20.9±3.1	-	-
33. Tarkci et al., 2022							
Control	93.4±27.4	-	-	-	-	160.6±2.6	-
HFD+kefir	71.1±40.3	-	-	-	-	120.9±2.2	-
34. Tung et al., 2020							
Control	-	410.0±2.5	450.0±4.0	21.0±2.0	-	-	-
HFD/ApoE$^{-/-}$ (+ KPs-L)	-	420.0±1.5	180.0±2.0	29.0±3.0	-	-	-
HFD/ApoE$^{-/-}$ (+ KPs-H)	-	340.0±3.0	150.0±1.0	38.0±3.0	-	-	-

Supplementary Table S7

Evaluating outcomes associated with metabolic syndrome parameters highlights the effects of kefir and its active components in rodent models.

Author, Year	Metabolic-related parameters (mean±SD)						
(Tested group)	WG (g)	TC (mg/dL)	TG (mg/dL)	LDL-C (mg/dL)	HDL-C (mg/dL)	PG (mg/dL)	PI (ng/mL)
35. Tung et al., 2018							
Control	71.2±1.0	53.6±0.9	158.6±5.8	11.2±6.1	19.1±0.5	-	-
HFD+KPs	20.2±1.2	61.3±2.4	164.3±4.7	6.3±0.2	19.1±0.8	-	-
36. Youn et al., 2022							
Control	8.5±0.5	119.0±3.5	123.0±17.1	9.1±0.4	70.2±1.5	143.0±19.6	0.71±0.06
HFD+LAB	7.0±0.4	114±7.3	96.2±8.8	9.6±0.6	66.0±2.3	141.0±16.7	0.75±0.07
HFD+CPB	4.8±0.3	127.0±7.0	82.9±12.0	10.9±1.0	75.1±4.8	128.0±17.3	0.71±0.04
37. Zheng et al., 2024							
Control	19.0±3.0	167.1±19.7	109.8±29.8	-	-	274.2±22.8	-
HFD+L-ABK	16.0±4.0	165.7±23.0	55.6±15.8	-	-	249.8±20.8	-
HFD+M-ABK	14.0±3.0	156.1±14.0	59.8±6.8	-	-	247.9±32.5	-
HFD+H-ABK	12.0±5.5	148.8±18.9	41.9±10.3	-	-	240.6±27.6	-
HFD+L-TWK	18.0±6.0	162.6±14.5	43.0±7.7	-	-	242.7±36.6	-
HFD+M-TWK	10.5±5.5	146.7±11.5	35.9±6.3	-	-	224.0±19.8	-
HFD+H-TWK	10.7±3.5	148.2±16.5	39.6±14.3	-	-	213.0±34.9	-

Supplementary Table S7

Evaluating outcomes associated with metabolic syndrome parameters highlights the effects of kefir and its active components in rodent models.

Author, Year	Metabolic-related parameters (mean±SD)						
(Tested group)	WG (g)	TC (mg/dL)	TG (mg/dL)	LDL-C (mg/dL)	HDL-C (mg/dL)	PG (mg/dL)	PI (ng/mL)
38. Zubiria et al., 2017							
Control	34.0±0.3	-	3.2±0.6	-	-	150.0±20.0	-
FRD+LK	33.2±0.3	-	1.5±0.2	-	-	130.0±15.0	-

* WG: Weight gain, TC: Total cholesterol, TG: Triglyceride, LDL-C: Low-density lipoprotein-cholesterol, HDL-C: High-density lipoprotein-cholesterol, VLDL-C: Very low-density lipoprotein-cholesterol, PG: Plasma glucose, PI: plasma insulin, HCLD: Hypercholesterolemic diet, HFD: High fat diet, C-Kefir: Commercial kefir, ICK, IR9, IR10, and GER2: Four traditional kefir gains, PK1, P-Kefir: A pitched kefir, CFK: A centrifuged and filter sterilized cell-free fraction, HK: A heat-treated fraction, PNL: A pitched kefir without the inclusion of *Lactobacillus* species, PNY: pitched kefir without the inclusion of yeast species, AD: atherogenic diet, KPs: Kefir peptides (KPs-L: A low dose of KPs and KPs-H: A high dose of KPs), CFM: commercial fermented milk, WD: Western diet, ABK: AB-Kefir from SYNBIO TECH INC (Kaohsiung, Taiwan), APS1: *Lactobacillus mali* APS1 at 5×10^7 CFU (APS1-L), 5×10^8 CFU (APS1-M) and 5×10^9 CFU (APS1-H), GSF: A prebiotic polyphenol-rich wine grape seed flour at 5% for GSFL or 10% for GSFH, LAB: lactic acid bacteria isolated from kefir, LK: a 0.1% (w/w) kefir powder-supplemented high-fat diet, HK: a 0.2% (w/w) kefir powder-supplemented high-fat diet, Lactic-F: Lactobacillus ferment, HFCS: High-fructose corn syrup, TKM: Tibet kefir milk, DH4: *Leuconostoc mesenteroides* DH4, DH5; *Lactobacillus kefiri* DH5, DH7; *Lactobacillus kefiri* DH7, SDH5: Surface layer proteins derived from kefir DH5, SLCM8: Surface layer proteins derived from kefir *Leuconostoc mesenteroides* DH1608, EPS: Water-soluble exopolysaccharides isolated from kefir, Res: residues after EPS removal from kefir, M1: *Lactobacillus kefiranofaciens*, APS1: *Lactobacillus mali* APS1, P-Kefir: Probiotic kefir, S-Kefir: Symbiotic kefir, SIM: simvastatin, HFFrD: High-fat and high-fructose diet, HLAB: Paraprobiotic heat-killed lactic acid bacteria, L-kefir: A low dose kefir, H-kefir: A high dose kefir, CPB: A product of citrus pomace extract and whey using kefir bacteria, TWK: *Lactiplantibacillus plantarum* TWK10, FRD: Fructose-rich diet, LK: *Lactobacillus kefiri*

Supplementary Table S8

Assessing outcomes related to inflammatory and oxidative stress markers highlights the effects of kefir and its active components in rodent models of metabolic syndrome.

Author, Year	Inflammatory and oxidative stress markers (mean±SD)				
(Tested group)	TNF-α (pg/mL)	IL-6 (pg/mL)	IL-1β (pg/mL)	MDA (μM)	ox-LDL (mg/ml)
1. Akar et al., 2021					
Control	26.0±4.0	-	38.9±2.0	-	-
Fructose	44.8±2.0	-	79.0±4.5	-	-
Fructose+kefir	31.0±3.0	-	55.0±2.5	-	-
7. Chang et al., 2023					
Control	7.5±0.4	-	-	368.5±50.4	0.04±0.02
AD+KPs-L	6.4±0.6	-	-	273.3±42.6	0.01±0.002
AD+KPs-H	6.5±0.8	-	-	261.5±62.9	0.02±0.002
20. Kim et al., 2017					
Control	560.0±4.5	115.0±8.0	485.0±3.5	-	-
Kefir	580.0±1.0	52.0±2.0	490.0±1.5	-	-
24. Lin et al., 2016					
Control	-	3.5±10.0	-	-	-
HFD+M1	-	0.8±0.2	-	-	-
HFD+APS1	-	1.0±0.8	-	-	-
26. Nurliyani et al., 2022					
Control	271.3±167.9	-	-	-	-
HFHF+P-kefir	208.0±44.7	-	-	-	-
HFHF+S-kefir	155.0±6.6	-	-	-	-
HFHF+SIM	192.3±50.5	-	-	-	-

Supplementary Table S8

Assessing outcomes related to inflammatory and oxidative stress markers highlights the effects of kefir and its active components in rodent models of metabolic syndrome.

Author, Year	Inflammatory and oxidative stress markers (mean±SD)				
(Tested group)	TNF-α (pg/mL)	IL-6 (pg/mL)	IL-1β (pg/mL)	MDA (μM)	ox-LDL (mg/mL)
28. Santanna et al., 2017					
Control	590.0±2.5	240.0±3.0	-	-	-
HFD+milk	350.0±1.5	280.0±5.0	-	-	-
HFD+kefir	545.0±2.0	150.0±1.0	-	-	-
34. Tung et al., 2020					
Control	70.1±2.5	-	46.0±2.5	-	-
HFD/ApoE$^{-/-}$ (+ KPs-L)	42.5±4.0	-	18.0±2.8	-	-
HFD/ApoE$^{-/-}$ (+ KPs-H)	28.5±0.8	-	5.0±0.2	-	-

*TNF-α: Tumor necrosis factor-alpha, IL-6: Interleukin-6, IL-1β: Interleukin-1beta, MDA: Malondialdehyde, ox-LDL: Oxidized low-density lipoprotein

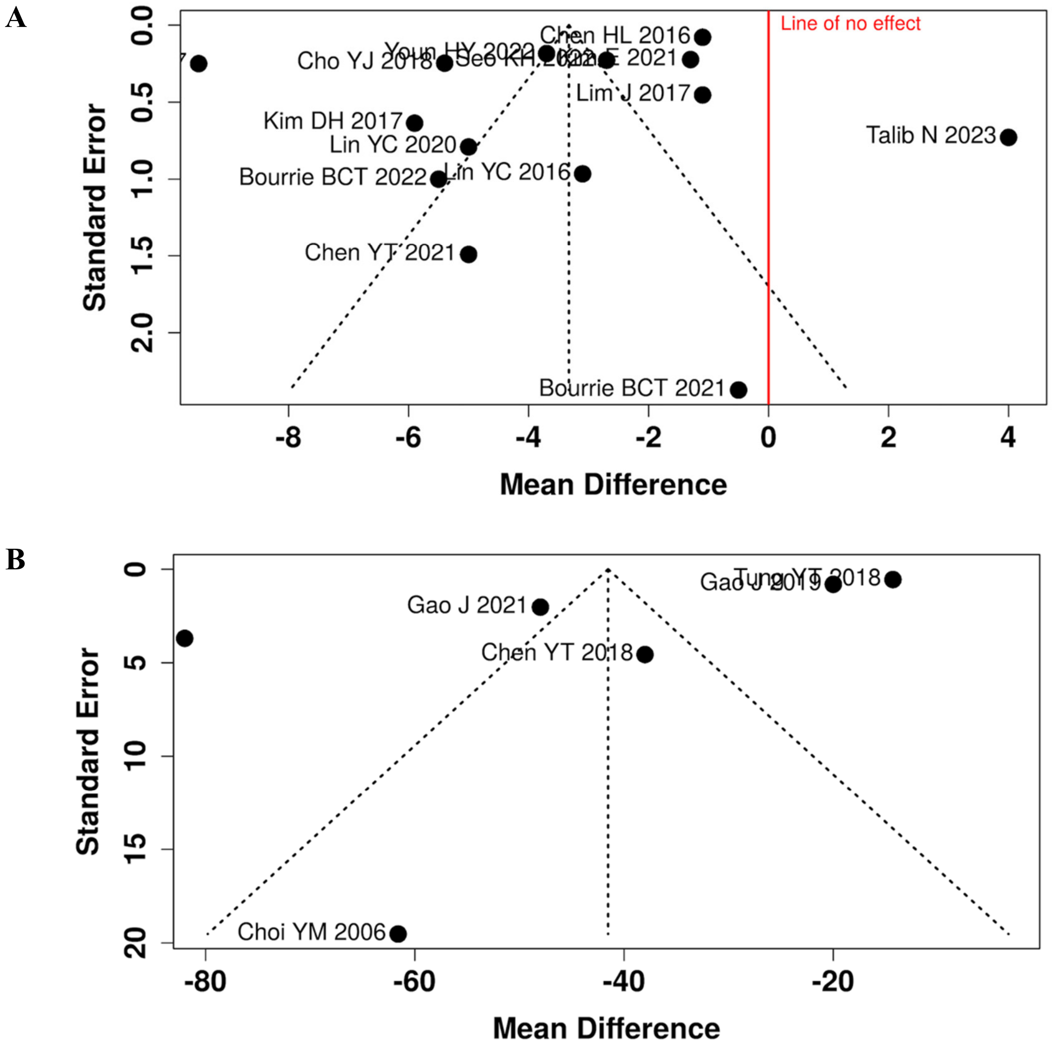

Supplementary Figure S1. A tunnel plot illustrating the distribution of publication biases associated with the consumption of various types of kefirs, their isolated bacteria, or active components, compared to the control group, regarding weight gain in mouse (A) and rat (B) models.

A

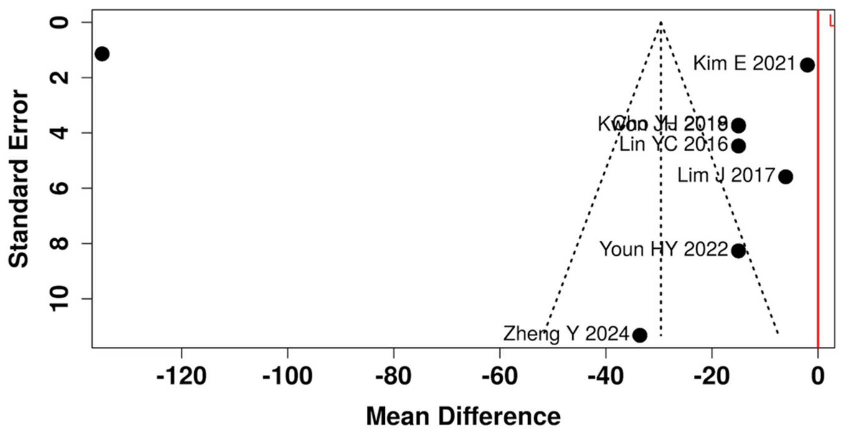

B

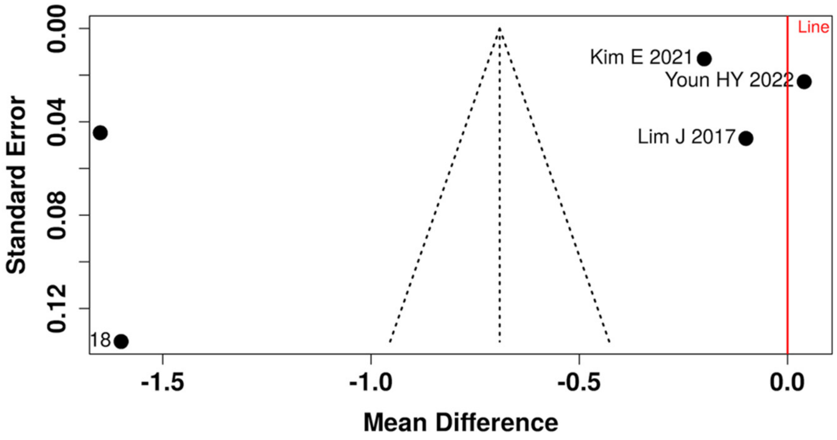

Supplementary Figure S2 A tunnel plot illustrating the distribution of publication biases associated with the consumption of various types of kefirs, their isolated bacteria, or active components, compared to the control group, on plasma glucose (A) and insulin (B) levels in mouse models.

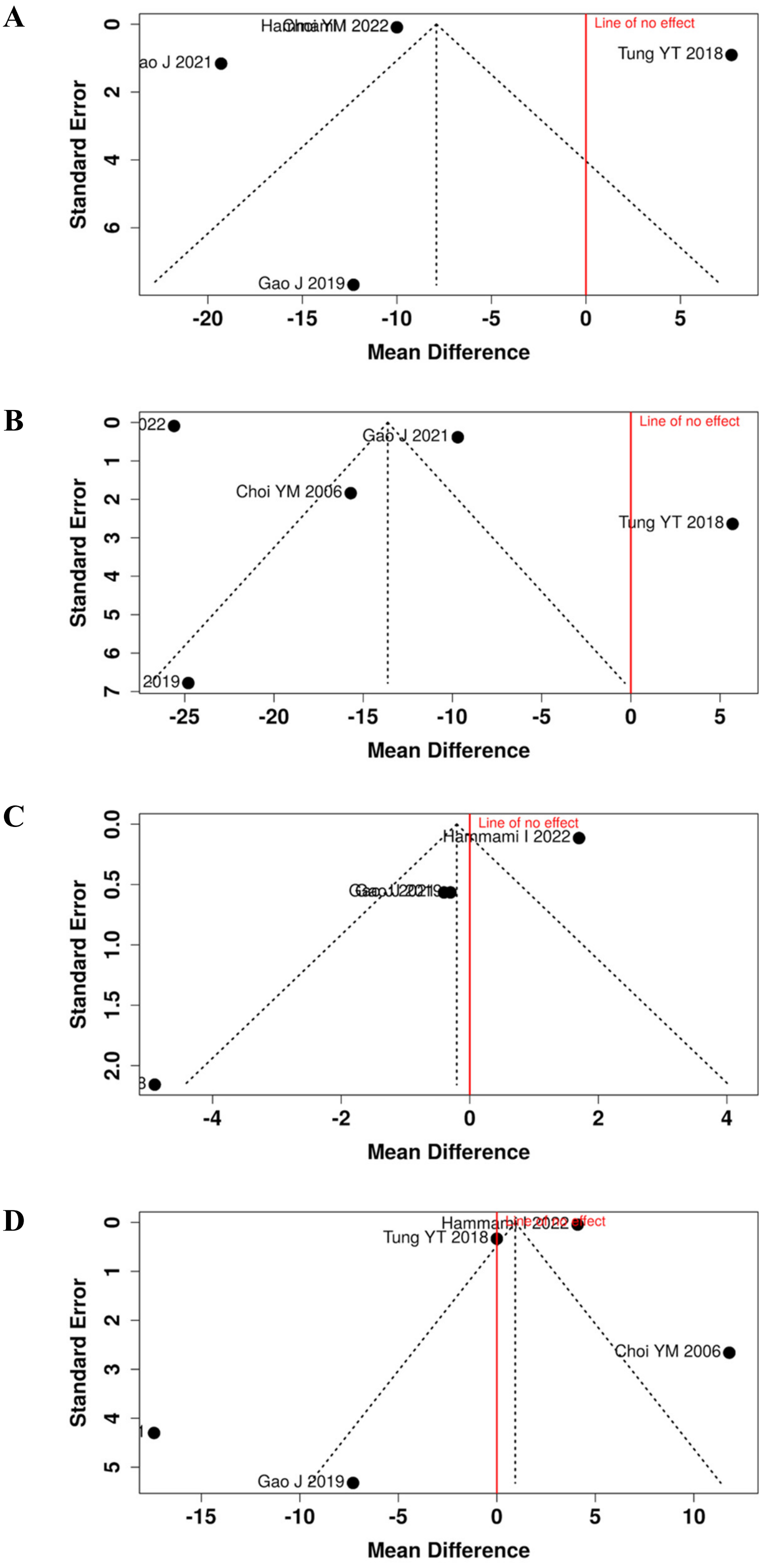
A
Standard Error
Mean Difference
Line of no effect
ao J 2021
Tung YT 2018
Gao J 2019
0
2
4
6
-20
-15
-10
-5
0
5
B
Standard Error
Mean Difference
Line of no effect
022
Gao J 2021
Choi YM 2006
Tung YT 2018
2019
0
1
2
3
4
5
6
7
-25
-20
-15
-10
-5
0
5
C
Standard Error
Mean Difference
Line of no effect
Hammami I 2022
0.0
0.5
1.0
1.5
2.0
-4
-2
0
2
4
D
Standard Error
Mean Difference
Tung YT 2018
Choi YM 2006
Gao J 2019
0
1
2
3
4
5
-15
-10
-5
0
5
10

Supplementary Figure S3. A tunnel plot illustrating the distribution of publication biases associated with the consumption of various types of kefirs, their isolated bacteria, or active components, in comparison to the control group, on lipid profiles, including levels of total cholesterol (A), triglycerides (B), low-density lipoprotein (C), and high-density lipoprotein (D) in rat models.

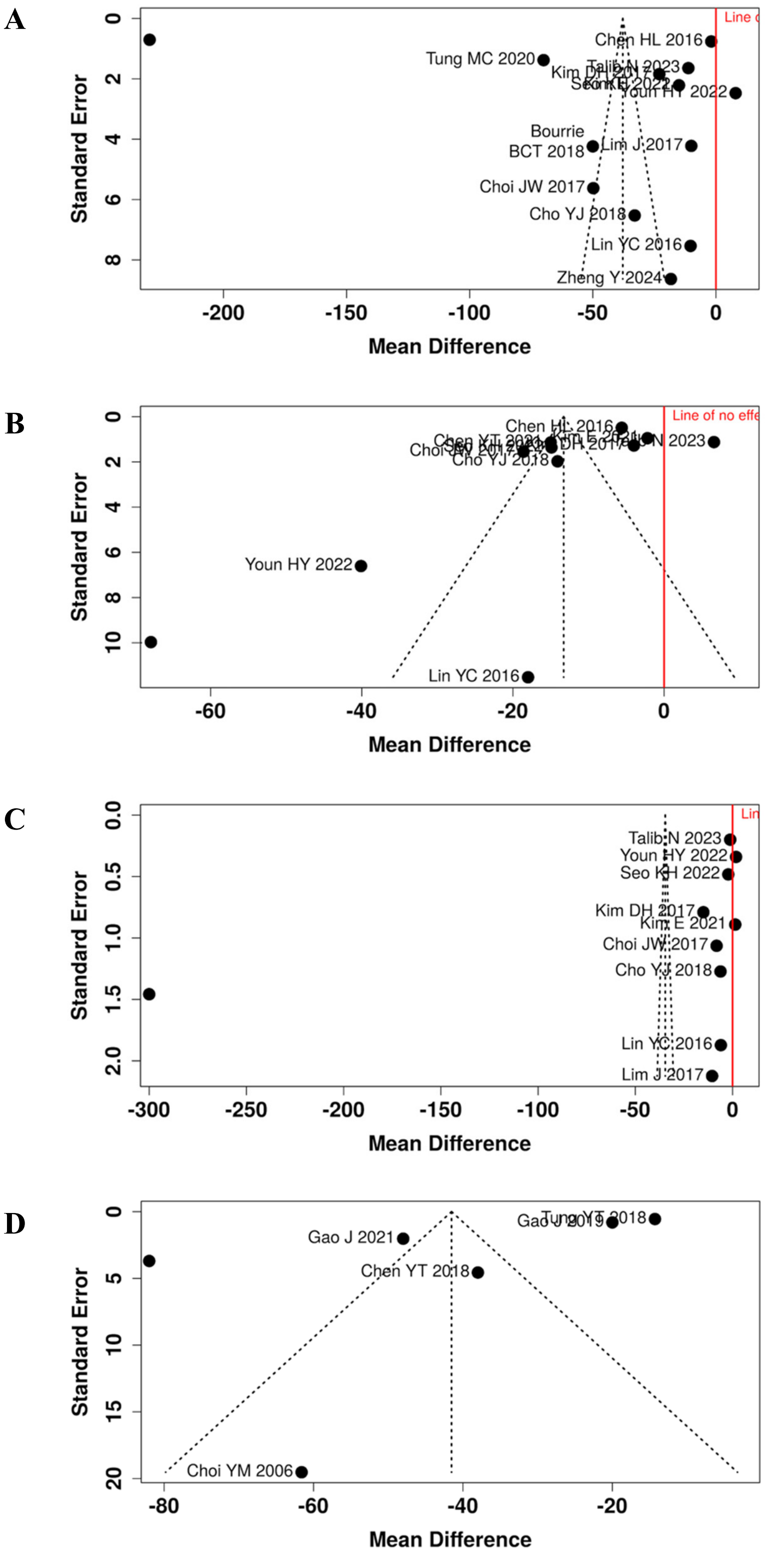
A
Standard Error
Mean Difference
Chen HL 2016
Tung MC 2020
Talib N 2023
Youn HY 2022
Bourrie
BCT 2018
Lim J 2017
Choi JW 2017
Cho YJ 2018
Lin YC 2016
Zheng Y 2024
B
Standard Error
Mean Difference
Line of no effe
Chen HL 2016
Cho YJ 2018
Youn HY 2022
Lin YC 2016
C
Standard Error
Mean Difference
Talib N 2023
Youn HY 2022
Seo KH 2022
Kim DH 2017
Kim E 2021
Choi JW 2017
Cho YJ 2018
Lin YC 2016
Lim J 2017
D
Standard Error
Mean Difference
Gao J 2021
Chen YT 2018
Choi YM 2006

Supplementary Figure S4. A tunnel plot illustrating the distribution of publication biases associated with the consumption of various types of kefirs, their isolated bacteria, or active components, in comparison to the control group, on lipid profiles, including levels of total cholesterol (A), triglycerides (B), low-density lipoprotein (C), and high-density lipoprotein (D) in mouse models.
